# Repeated fed-batch strategy and metabolomic analysis to achieve high docosahexaenoic acid productivity in *Crypthecodinium cohnii*

**DOI:** 10.1186/s12934-020-01349-6

**Published:** 2020-04-16

**Authors:** Liangsen Liu, Fangzhong Wang, Guangsheng Pei, Jinyu Cui, Jinjin Diao, Mingming Lv, Lei Chen, Weiwen Zhang

**Affiliations:** 1 Laboratory of Synthetic Microbiology, School of Chemical Engineering & Technology, Tianjin University, Tianjin, 300072 People’s Republic of China; 2 Frontier Science Center for Synthetic Biology and Key Laboratory of Systems Bioengineering (MOE), School of Chemical Engineering and Technology, Tianjin University, Tianjin, 300350 People’s Republic of China; 3 Center for Biosafety Research and Strategy, Tianjin University, Tianjin, People’s Republic of China; 4 SynBio Research Platform, Collaborative Innovation Center of Chemical Science and Engineering (Tianjin), Tianjin, People’s Republic of China

**Keywords:** Repeated fed-batch culture, Docosahexaenoic acid, Productivity, Metabolomic, Stability, *Crypthecodinium cohnii*

## Abstract

**Background:**

Docosahexaenoic acid (DHA) is essential for human diet. However, high production cost of DHA using *C*. *cohnii* makes it currently less competitive commercially, which is mainly caused by low DHA productivity. In recent years, repeated fed-batch strategies have been evaluated for increasing the production of many fermentation products. The reduction in terms of stability of culture system was one of the major challenges for repeated fed-batch fermentation. However, the possible mechanisms responsible for the decreased stability of the culture system in the repeated fed-batch fermentation are so far less investigated, restricting the efforts to further improve the productivity. In this study, a repeated fed-batch strategy for DHA production using *C*. *cohnii* M-1-2 was evaluated to improve DHA productivity and reduce production cost, and then the underlying mechanisms related to the gradually decreased stability of the culture system in repeated fed-batch culture were explored through LC– and GC–MS metabolomic analyses.

**Results:**

It was discovered that glucose concentration at 15–27 g/L and 80% medium replacement ratio were suitable for the growth of *C. cohnii* M-1-2 during the repeated fed-batch culture. A four-cycle repeated fed-batch culture was successfully developed and assessed at the optimum cultivation parameters, resulting in increasing the total DHA productivity by 26.28% compared with the highest DHA productivity of 57.08 mg/L/h reported using *C*. *cohnii*, including the time required for preparing seed culture and fermentor. In addition, LC– and GC–MS metabolomics analyses showed that the gradually decreased nitrogen utilization capacity, and down-regulated glycolysis and TCA cycle were correlated with the decreased stability of the culture system during the long-time repeated fed-batch culture. At last, some biomarkers, such as Pyr, Cit, OXA, FUM, l-tryptophan, l-threonine, l-leucine, serotonin, and 4-guanidinobutyric acid, correlated with the stability of culture system of *C. cohnii* M-1-2 were identified.

**Conclusions:**

The study proved that repeated fed-batch cultivation was an efficient and energy-saving strategy for industrial production of DHA using *C. cohnii*, which could also be useful for cultivation of other microbes to improve productivity and reduce production cost. In addition, the mechanisms study at metabolite level can also be useful to further optimize production processes for *C. cohnii* and other microbes.
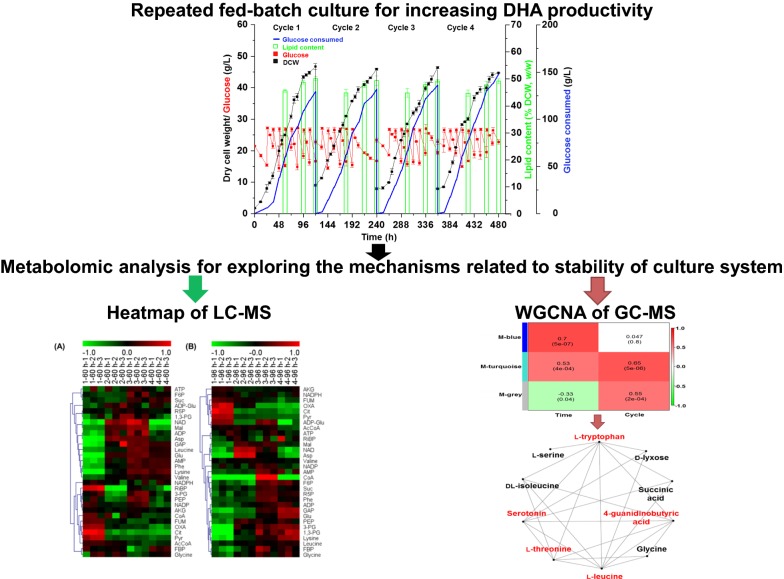

## Background

Docosahexaenoic acid (DHA) that is essential for human diet, a long chain polyunsaturated fatty acids (PUFAs) of *ω*-3 family, is an important structure component of human brain and retina [1]. Clinical studies showed that DHA also has positive effects on preventing some diseases, such as hypertension, atherosclerosis and some cancers [2]. The traditional source of DHA is fish oils. However, due to ocean pollution and sharp decrease of fish resources in recent decades, fermentation of heterotrophic microalgae has been proposed to be a promising alternative for DHA production [3, 4]. *Crypthecodinium cohnii*, a DHA-rich heterotrophic marine microalgae, has received great interest since it contains more than 50% lipid content in its dry cell weight (DCW) [3, 5]; and the ratio of DHA in total fatty acids (TFAs) approaches 50% [5]; furthermore, it does not contain any eicosapentaenoic acid (EPA) that is not suitable for infant use [6]. In recent years, *C. cohnii* has been widely used for industrial production of DHA in many countries [3, 7].

One of major issues for industrial production of DHA using *C*. *cohnii* is the high production cost, which is caused by low DHA productivity. Significant efforts have been made in the past to improve the DHA productivity in *C*. *cohnii* by fed-batch cultures [3, 8]. For example, 48 mg/L/h and 53 mg/L/h of DHA productivity were obtained using acetic acid and ethanol as the carbon source, respectively [8, 9]. The DHA productivity was further elevated to 57.08 mg/L/h using glucose as the carbon source [3]. However, for the purpose of the industrial production, elevation of DHA productivity by fed-batch culture alone may not be enough. According to recent studies [10, 11], repeated fed-batch strategy not only retains the advantages of fed-batch culture, such as relieving substrate inhibition, but also has many other distinctive characteristics. For example: (i) it saves the time and labor costs for seed preparation, cleaning and sterilization of the fermentor [10, 12]; (ii) it alleviates the inhibition of by-products, which typically accumulate to high contents during the stationary or late phases of batch and fed-batch fermentation [13]; (iii) it is beneficial to the stable downstream processes of product separation and purification [11]. In fact, in recent years repeated fed-batch strategies have been evaluated for increasing the production of many products [11, 14, 15]. For example, the production of the recombinant human serum albumin in yeast *Pichia pastoris* was enhanced by 47% through a repeated fed-batch fermentation strategy [11]. The cellulase productivity using *P. oxalicum* RE-10 was improved by 49.77% in a repeated fed-batch fermentation, compared with its batch fermentation [15]. The arachidonic acid (ARA) production period was shortened by 35% in a repeated fed-batch fermentation, compared to its fed-batch culture [14]. Therefore, these studies demonstrated that repeated fed-batch cultivation could be one of the promising measures to improve productivity and decrease fermentation cost of DHA production using *C. cohnii*.

As repeated fed-batch cultivation process typically lasts long time, the stability of culture system is an important factor [14, 16]. However, the stability of culture system is usually decreased as the cycle number increased, which could lead to fermentation stop, affecting total productivity negatively. For example, a repeated fed-batch fermentation of lipid production using *R*. *toruloides* lasted only 2 cycles, and then the lipid productivity was significantly decreased in cycle 3 [17]; the lutein productivity started to decline in cycle 4 because DCW was decreased, during the repeated fed-batch culture of *Desmodesmus* sp. F51 [16]; the production of ARA by *M*. *alpina* stopped after four cycles, because the ARA percentage in TFAs was obviously decreased [14]. These early studies suggested that the reduction of the stability of culture system was one of the major challenges for repeated fed-batch fermentation. However, the possible mechanisms responsible for the decreased stability of culture system in the repeated fed-batch fermentation are so far less investigated, restricting the efforts to further improve the productivity.

Metabolomic analysis is able to provide a metabolite-level representation of the phenotype for organisms due to its capability to detect a large array of small molecule metabolites qualitatively and quantitatively [18, 19]. In recent years, both liquid chromatography mass spectrometry (LC–MS) and gas chromatography mass spectrometry (GC–MS) based metabolomics have been widely applied to explore biology of various microalgae, including *C. cohnii* [20–22]. For example, the lipid accumulation mechanisms of *C*. *cohnii* responded to chemical modulators were explored using LC–MS based metabolomic analysis [21]; the glucose tolerance mechanisms of *C. cohnii* mutants obtained by adaptive laboratory evolution were analyzed by using GC–MS metabolomic analysis [22]; and the mechanisms of antioxidant on elevating lipid accumulation in *C. cohnii* were analyzed using integrated LC- and GC–MS metabolomic analyses [20]. Therefore, metabolomic analysis is considered to be a promising approach to study the changes in metabolites of *C. cohnii* during the long-time repeated fed-batch cultivation.

In this study, a repeated fed-batch fermentation strategy for efficient DHA production with *C. cohnii* was developed and evaluated by optimizing the parameters substrate concentration and medium replacement ratio. Metabolomic analysis was then used to explore the underlying mechanisms related to the reduced stability of culture system of *C. cohnii* during the repeated fed-batch fermentation process. This study provided an effective strategy for industrial production of DHA using *C*. *cohnii*, and new insights for maintaining the stability of culture system during the extended fermentation process.

## Results and discussion

### Effects of glucose concentration on DHA production in repeated fed-batch cultivation

**Fig. 1:**
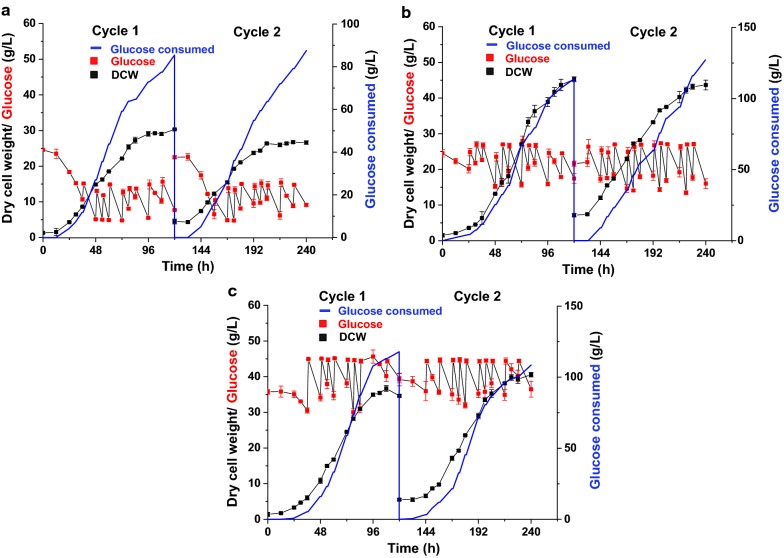
Repeated fed-batch cultivation of two cycles under different glucose concentrations at 25 °C and pH 6.5. **a** Repeated fed-batch culture with initial glucose monohydrate concentration 27 g/L and maintenance concentration 5–15 g/L of glucose; **b** Repeated fed-batch culture with initial glucose monohydrate concentration 27 g/L and maintenance concentration 15–27 g/L of glucose; **c** Repeated fed-batch culture with initial glucose monohydrate concentration 45 g/L and maintenance concentration 27–45 g/L of glucose

Our previous study showed that the specific growth rate and specific glucose consumption rate of *C. cohnii* M-1-2 were both decreased and maintained at a low rate after 120 h in fed-batch culture with initial inoculated volume at 10% (*v*/*v*) [3]. Therefore, the fermentation broth was initially replaced at 120 h of each cycle with replacement ratio at 90% (*v*/*v*). The concentration of carbon sources plays an important role in microbial metabolism, including *C. cohnii* [3, 8, 9, 14]. Therefore, the effect of glucose maintenance concentration on DHA production was first investigated in repeated fed-batch cultivation of *C. cohnii* M-1-2 (Fig. 1 and Table 1). The results showed that, for glucose concentration at 5–15 g/L, *C. cohnii* M-1-2 entered into stationary growth phase at 96 h in each cycle, and achieved the lowest DCW and DHA productivity of each cycle; for glucose concentration at 27–45 g/L, *C. cohnii* M-1-2 also entered into stationary growth phase at 96 h of each cycle; for glucose concentration at 15–27 g/L, *C. cohnii* M-1-2 kept growing and did not enter into stationary growth phase in both cycles during 120 h, and eventually achieved the highest DCW and DHA productivity in both cycles (Table 1). In addition, the DHA yield on glucose at 15–27 g/L was 47.61 and 40.42 mg/g for the two cycles, respectively, which was higher than the 39.92 and 34.15 mg/g on glucose at 5–15 g/L of the same two cycles. The DHA yield on glucose at 27–45 g/L was 39.16 mg/g in cycle 2, which was higher than 33.08 mg/g in cycle 1. Together, the results demonstrated that glucose concentration at 15–27 g/L was relatively suitable for the growth of *C. cohnii* M-1-2 during the repeated fed-batch cultivation.

**Table 1:** Comparison of DHA production at the end of each cycle using different glucose concentrations

Glucose Conc. (g/L)	Cycle	Fermentation time (h)	Dry cell weight (g/L)	DHA (g/L)	DHA productivity^a^ (mg/L/h)
5.00–15.00	Cycle 1	120	30.38 ± 0.12	3.41 ± 0.03	28.44 ± 0.24
5.00–15.00	Cycle 2	120	26.72 ± 0.49	2.99 ± 0.01	24.93 ± 0.10
15.00–27.00	Cycle 1	120	45.17 ± 0.71	5.42 ± 0.02	45.14 ± 0.15
15.00–27.00	Cycle 2	120	43.57 ± 1.37	5.14 ± 0.04	42.84 ± 0.30
27.00–45.00	Cycle 1	120	34.69 ± 0.35	3.90 ± 0.01	32.54 ± 0.12
27.00–45.00	Cycle 2	120	40.62 ± 0.63	4.25 ± 0.03	35.40 ± 0.25

^a^DHA productivity of each cycle (the DHA produced at the end of each cycle/the time of each cycle operation)

As mentioned above, glucose concentration maintained at 15–27 g/L was more suitable for *C. cohnii* M-1-2 growth in comparison with that at 27–45 g/L, consistent with the previous reports that high glucose concentration inhibited the growth of *C. cohnii* ATCC 30556 or 30772 in batch culture [23, 24], in which the highest specific growth rate and DCW were achieved at an initial glucose concentration 20 g/L of *C. cohnii* ATCC 30556 [23], and the initial growth rate of *C. cohnii* ATCC 30772 was reduced when the initial glucose concentration was above 25 g/L [24]. The glucose inhibition on growth was also observed in other lipid producing microbes [17]. For example, the DCW of *R. toruloides* Y4 were increased by 37.8% at 5 g/L of glucose concentration, compared with glucose concentration maintained at 30 g/L [17]. High glucose concentration of 15–27 and 27–45 g/L was found to inhibit the DHA synthesis significantly in cycle 2 compared with that in cycle 1 (Fig. 3a). The inhibition to DHA synthesis by high glucose was also found in other *C. cohnii* studies [22, 23]. For example, the DHA percentage of TFAs in *C*. *cohnii* ATCC 30556 was reduced to 44.7% from 53.4%, when glucose concentration was increased to 20 g/L from 5 g/L [23]. The total lipid in DCW and DHA percentage of TFAs in *C. cohnii* ATCC 30556 were all gradually decreased with the increase of glucose concentration from 9 to 54 g/L [22]. One possible reason of high glucose inhibition might be the increased osmotic pressure of the culture medium, which results in more energy consumption required for cellular maintenance [25]. A higher initial number of cells was found to increase the stress adaptation of *Escherichia coli* [26]. The final DCW of cycle 2 with glucose concentration at 27–45 g/L was increased by 17.09%, compared with that of cycle 1 with glucose concentration at 27–45 g/L, which might be due to that the initial number of cells in the cycle 2 of 27–45 g/L was much larger than that in the cycle 1 of 27–45 g/L, resulting in enhancing the resistance of *C. cohnii* M-1-2 to high glucose inhibition.

### Effects of medium replacement ratio on DHA production in repeated fed-batch cultivation

Medium replacement ratio not only affects the composition of the fermentation broth, but also affects the inoculated cell number. Therefore, the medium replacement ratio was further optimized (Table 2). The results showed that DCW of 80% medium replacement ratio was increased by 11.15% and 5.21%, compared with 70% and 90% medium replacement ratio, respectively. The DHA content in DCW (*w/w*) of 80% medium replacement ratio was elevated by 12.64% in comparison with 70% medium replacement ratio, but it remained at the similar level with 90% medium replacement ratio (Fig. 3b). The DHA productivity was the highest at 80% medium replacement ratio, which was 25.33% and 5.70% higher than 70% and 90% medium replacement ratios, respectively. Therefore, 80% medium replacement ratio was selected for the repeated fed-batch cultivation of *C*. *cohnii* M-1-2.

**Table 2:** Comparison of DHA production by repeated fed-batch culture using different medium replacement ratios

Replacement ratio (%)	Cycle	Fermentation time (h)	Dry cell weight (g/L)	DHA (g/L)	DHA productivity (mg/L/h)
90^a^	Cycle 2	120	43.57 ± 1.37	5.14 ± 0.04	42.84 ± 0.30
80	Cycle 2	120	45.84 ± 0.52	5.43 ± 0.02	45.28 ± 0.13
70	Cycle 2	120	41.24 ± 0.78	4.34 ± 0.15	36.13 ± 1.29

^a^The results were from Table 1

The DCW, DHA content in DCW (*w/w*) and DHA productivity of cycle 2 were all decreased at 70% medium replacement ratio compared with those at 80% medium replace ratio of cycle 2 (Table 2 and Fig. 3b). It was previously known that *C. cohnii* produced a large amount of extracellular polysaccharides at the late stage of the fermentation process, which increased the viscosity of fermentation broth and reduced the efficiency of oxygen transfer, leading to a low DHA production [27]. Therefore, we speculated that extra-metabolites or by-products secreted by *C*. *cohnii* and possible accumulation of unspent medium components might affect the growth and DHA synthesis, resulting in inhibition of the growth and DHA synthesis at 70% medium replacement ratio. When the replacement ratio was maintained at 80%, the inhibition was efficiently relieved [28]. Medium replacement ratio was also found as an important parameter in several other studies in repeated fed-batch culture [12, 15]. For example, the 80% medium replacement ratio was found to be the optimized culture condition for *Arthrospira* (*Spirulina*) *platensis* in the repeated fed-batch culture compared with 50% or 95% [29]. The DHA productivity of *Schizochytrium* sp. was increased by 27.84% at 80% medium replacement ratio compared with 95% in repeated fed-batch culture [12]. However, 50% medium replacement ratio was found to be the most suitable for the production of cellulase by *Penicillium oxalicum* RE-10 [15], and 50% medium replacement ratio was also used to achieve the best production of xylitol by *Candida magnoliae* TISTR 5663 [30]. One of the possible reasons for the difference in the optimal medium replacement ratio might be related to the types of microbes and products [15, 31].

### DHA production in repeated fed-batch cultivation with multiple cycles

**Fig. 2:**
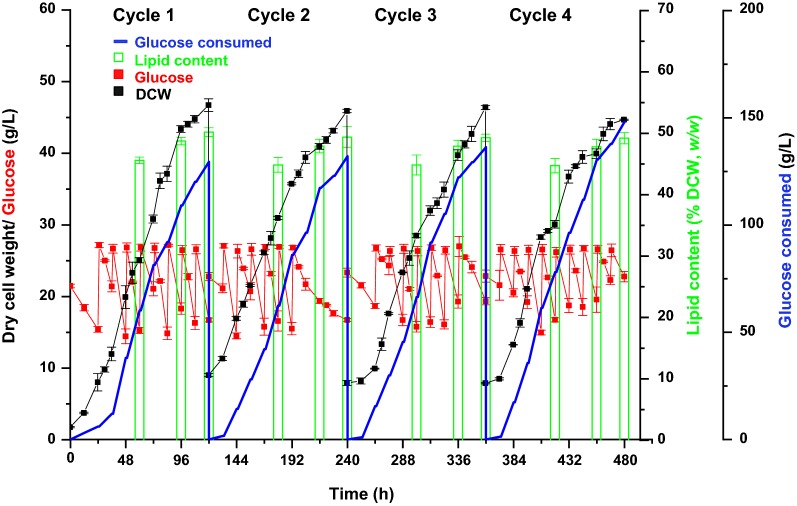
Repeated fed-batch cultivation of four cycles for DHA production at 25 °C and pH 6.5

**Fig. 3:**
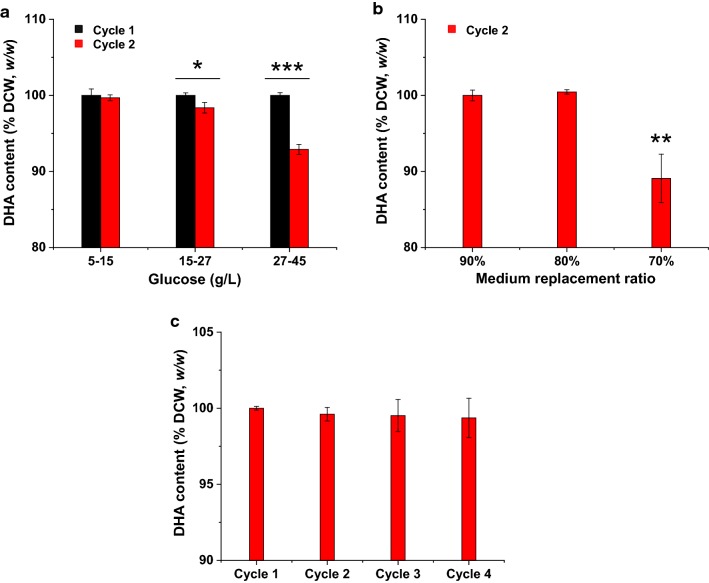
DHA content (% DCW, *w/w*) in repeated fed-batch cultivation of two and four cycles. **a** DHA content of two cycles under different glucose concentrations. The DHA content in cycle 1 was set as 100% under different glucose concentrations respectively, and the percentage changes in cycle 2 were shown; **b** DHA content of two cycles under different medium replacement ratios. The DHA content in cycle 2 under medium replacement ratio of 90% was set as 100%, and the percentage changes in cycle 2 under medium replacement ratio of 80% and 70% were shown; **c** DHA content of four cycles. The DHA content in cycle 1 was set as 100%, and the percentage changes in other cycles were shown. Asterisks indicated significant differences, as evaluated by a statistical Student’s *T*-test (*p < 0.05, **p < 0.01, ***p < 0.001)

After optimizing glucose concentration and medium replacement ratio in repeated fed-batch culture with two cycles, efforts were made to achieve more cycles (Fig. 2). For the repeated fed-batch culture with multiple cycles, the initial inoculation volume was set as 20% (*v/v*) and the medium replacement ratio was 80% (*v/v*) with glucose concentration maintained at 15-27 g/L, respectively. The results showed that the DCW were at the similar level of both cycle 2 and cycle 3 compared with that of cycle 1, but it was reduced significantly by 4.36% of cycle 4 compared with that of cycle 1, as demonstrated by a statistical analysis (Table 3). The DHA content in DCW (*w/w*) remained the similar level through all four cultivation cycles (Fig. 3c). The results suggested that growth rather than the DHA synthesis of *C. cohnii* M-1-2 started to be inhibited at cycle 4 (Table 3 and Fig. 3c). Moreover, the DHA yield on glucose of each cycle were 47.72, 45.80, 44.82, and 39.70 mg/g respectively with a 16.81% drop between cycle 1 and cycle 4, which was due to a 16.67% drop of DCW yield on glucose between cycle 1 and cycle 4. In addition, the color of fermentation broth gradually became dark mainly due to cell mass with the cycle number increased (data not shown), which also suggested cell state might be changed [14]. Therefore, the repeated fed-batch culture of *C. cohnii* M-1-2 was stopped after cycle 4. Table 4 showed the DHA productivity by *C*. *cohnii* using different fermentation strategies. The analysis showed that only two DHA productivities using *C*. *cohnii* [3, 9], were slightly higher than each individual cycle of the repeated fed-batch culture, so the DHA productivity of each individual cycle in this study was all maintained at a relatively high level compared with other fermentation strategies. Furthermore, the total productivity of repeated fed-batch culture with 4 cycles was increased by 26.28% compared with the reported highest DHA productivity of 57.08 mg/L/h in *C*. *cohnii* considering the time for preparing seed culture and fermentor, suggesting its application advantages [3, 14]. What’s more, the lipid content in DCW (*w/w*) and fatty acids profiles both showed no changes, which suggested that the quality of our products was stable during the repeated fed-batch with 4 cycles (Fig. 2 and Table 3). Together, a repeated fed-batch strategy to achieve high DHA productivity in *C. cohnii* was successfully established.

**Table 3:** Comparison of DHA production of each cycle in repeated fed-batch culture of four cycles

Repeated fed-batch	Fermentation time (h)	Dry cell weight (g/L)	DHA (g/L)	DHA productivity (mg/L/h)	Fatty acid composition (%)^a^					
					C12:0	C14:0	C16:0	C18:0	C18:1	C22:6
Cycle 1	120	46.80 ± 0.87	6.18 ± 0.01	51.54 ± 0.06	3.08 ± 0.36	14.28 ± 1.13	18.77 ± 0.56	2.58 ± 0.36	12.18 ± 0.18	49.12 ± 2.04
Cycle 2	120	45.98 ± 0.24	6.05 ± 0.03	50.43 ± 0.22	3.74 ± 0.26	14.00 ± 0.20	19.45 ± 0.18	2.19 ± 0.03	11.61 ± 0.23	49.02 ± 0.50
Cycle 3	120	46.48 ± 0.29	6.11 ± 0.06	50.94 ± 0.54	3.88 ± 0.04	12.95 ± 0.84	19.16 ± 0.93	2.85 ± 0.36	12.09 ± 0.36	49.06 ± 1.48
Cycle 4	120	44.76 ± 0.08^*^	5.88 ± 0.08	48.97 ± 0.64	3.58 ± 0.35	12.82 ± 0.58	20.25 ± 0.38	3.43 ± 0.73	11.40 ± 0.34	48.53 ± 0.49

^a^Lauric acid (C12:0). Myristic acid (C14:0). Palmitic acid (C16:0). Stearic acid (C18:0). Oleic acid (C18:1). Docosahexaenoic acid (C22:6)

* Significant differences between cycle 4 and cycle 1 evaluated by a statistical Student’s *T*-test (*p < 0.05)

**Table 4:** Comparison of DHA productivity using *C. cohnii* by different culture strategies

Culture strategy	*Crypthecodinium cohnii*	Carbon source	Fermentation time (h)	DHA productivity (mg/L/h)	References
Fed-batch	CCMP 316	Glucose	135	9.78	[48]
Batch	ATCC 30772	Glucose	91	17.58	[24]
Fed-batch	CCMP 316	Carob pulp	100	19.00	[49]
Fed-batch	ATCC 30556	Date syrup	240	23.54	[7]
Fed-batch	ATCC 30772	Acetic acid	400	47.50	[8]
Fed-batch	ATCC 30772	Ethanol	220	53.18	[9]
Fed-batch	M-1-2	Glucose	168	57.08	[3]
Repeated fed-batch cycle 1	M-1-2	Glucose	120	51.54	This study
Repeated fed-batch cycle 2	M-1-2	Glucose	120	50.43	This study
Repeated fed-batch cycle 3	M-1-2	Glucose	120	50.94	This study
Repeated fed-batch cycle 4	M-1-2	Glucose	120	48.97	This study

Seed culture is important for the final DCW and DHA productivity of *C. cohnii*, but it took much time and efforts to prepare seed culture for fed-batch fermentation [3, 8, 9, 24]. Repeated fed-batch fermentation only needs inoculation once and saves time on cleaning and sterilization of fermenter, greatly improved the productivity [14]. In addition, the costs of staffs, seed raw materials, and the energy consumption for cleaning and sterilization of fermenter could be apparently reduced [12]. Therefore, repeated fed-batch culture was a good option to reduce the cost of DHA production in *C. cohnii*, especially for large-scale industrial production. Repeated fed-batch culture has also been used successfully in the culture of many other microorganisms, such as *P*. *oxalicum* RE-10 and *C*. *parapsilosis* [15, 32].

As mentioned above (Table 3), the DCW and DCW yield on glucose of *C*. *cohnii* M-1-2 were both decreased significantly at the cycle 4 compared with those of cycle 1, and the color of fermentation broth was gradually changed,resulting in the fermentation stopping after cycle 4. Therefore, the stability of the culture system seemed gradually decreased as cycle number increased. It has been demonstrated that 70% replacement ratio can result in the inhibition to *C*. *cohnii* M-1-2 (Table 2). Therefore, it was reasonable to speculate the possible accumulation of inhibition compounds in the medium either from the growth medium or secreted by the cells, and the possible changed cell stability might be the reasons for the changes about the stability of culture system as cycle number increased and then the DCW of *C*. *cohnii* M-1-2 would be affected negatively [31, 33]. Similar phenomenon was also observed in other studies during the repeated fed-batch culture [14, 33].

### LC–MS based metabolomic analysis of the repeated fed-batch culture with 4 cycles

**Fig. 4:**
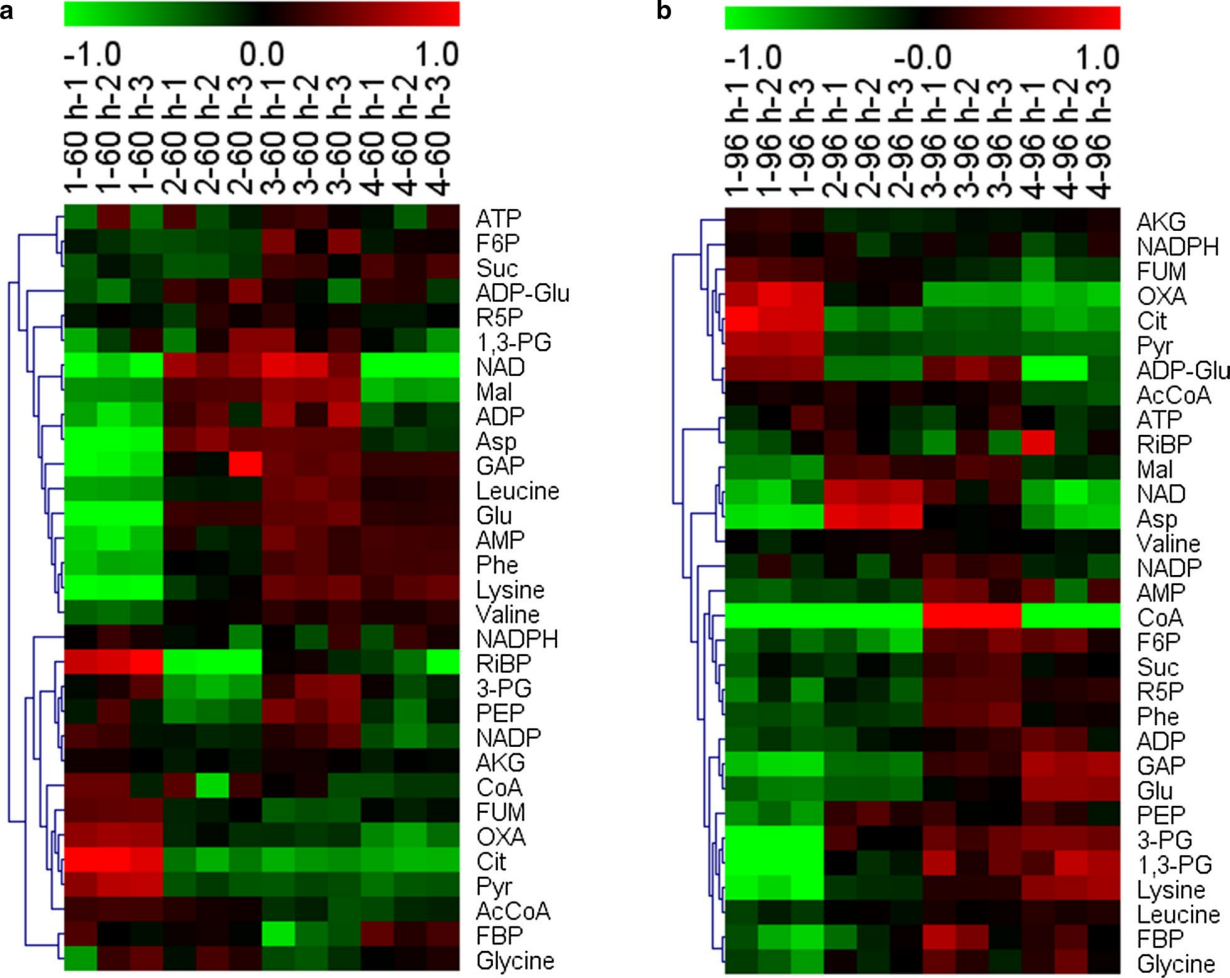
Heatmaps of LC–MS metabolomic profiles of repeated fed-batch cultivation with 4 cycles. **a** 60 h; **b** 96 h. The first number represents the number of cycles and the third number represents biological replicates of 1–60 h-1

LC–MS metabolomic analysis aiming mainly at unstable metabolites of central carbohydrate and energy metabolism [20] have been well established to probe the physiological change of *C. cohnii* M-1-2 [20, 21]. Cell samples collected at 60 and 96 h of each cycle, which were both in the linear growth phases, were subjected to LC–MS based metabolic analysis to explore possible mechanisms related to the changes about the stability of culture system during repeated fed-batch culture of *C. cohnii* M-1-2. As shown in Fig. 4, the content of metabolites was changed gradually as the cycle number increased, including: (i) intracellular abundances of amino acids, such as valine, leucine, glutamic acid, lysine, and phenylalanine at 60 h, and glycine, leucine, glutamic acid, lysine, and phenylalanine at 96 h, were constantly up-regulated as the cycle progressed (Fig. 4). *C. cohnii* M-1-2 was known to accumulate large amounts of amino acids under high nitrogen condition [3]. Continuous up-regulation of these amino acids suggested that the nitrogen utilization capacity of *C. cohnii* M-1-2 might be constantly decreased as cycle number increased; (ii) the continuous up-regulation of PEP, 3-PG, 1,3-PG, GAP, F6P, and FBP at 96 h, and the continuous up-regulation of GAP at 60 h was observed. However, Pyr at 60 and 96 h was both gradually down-regulated, suggesting that the metabolic flux from glycolysis pathway was gradually decreased as cycle number increased; (iii) the continuous down-regulation of Cit, OXA, and FUM at 60 h, and the continuous down-regulation of Cit, OXA, FUM, and AKG at 96 h suggested that tricarboxylic acid (TCA) cycle might be gradually attenuated as the cycle number increased. Therefore, the up-regulation of amino acids might consume the carbon skeletons of a large number of organic acids from TCA cycle, which might lead to the down-regulation of TCA cycle, reducing the cell activity of *C. cohnii* M-1-2 [3, 34]. Down-regulated TCA cycle has also been found in other aging organisms and cells, such as *Drosophila melanogaster* and testicular [35, 36]. It was interesting to find that the changes of metabolites above had two distinct characteristics. One was that the changes between the two adjacent cycles were small, but it was large between the cycle 1 and cycle 4. Another was that the change direction of the same metabolites was consistent among different cycles. The possible reasons that only the DCW of cycle 4 decreased compared with that of cycle 1 might be that certain degrees of changes were required to affect the stability of culture system negatively. In summary, these analyses suggested that the gradually decreased nitrogen utilization capacity, the continuous down-regulated glycolysis and TCA cycle might be the key factors that affected the stability of culture system of *C*. *cohnii* M-1-2 during the repeated fed-batch culture with multiple cycles.

### GC–MS based metabolomic analysis of the repeated fed-batch culture with 4 cycles

**Fig. 5:**
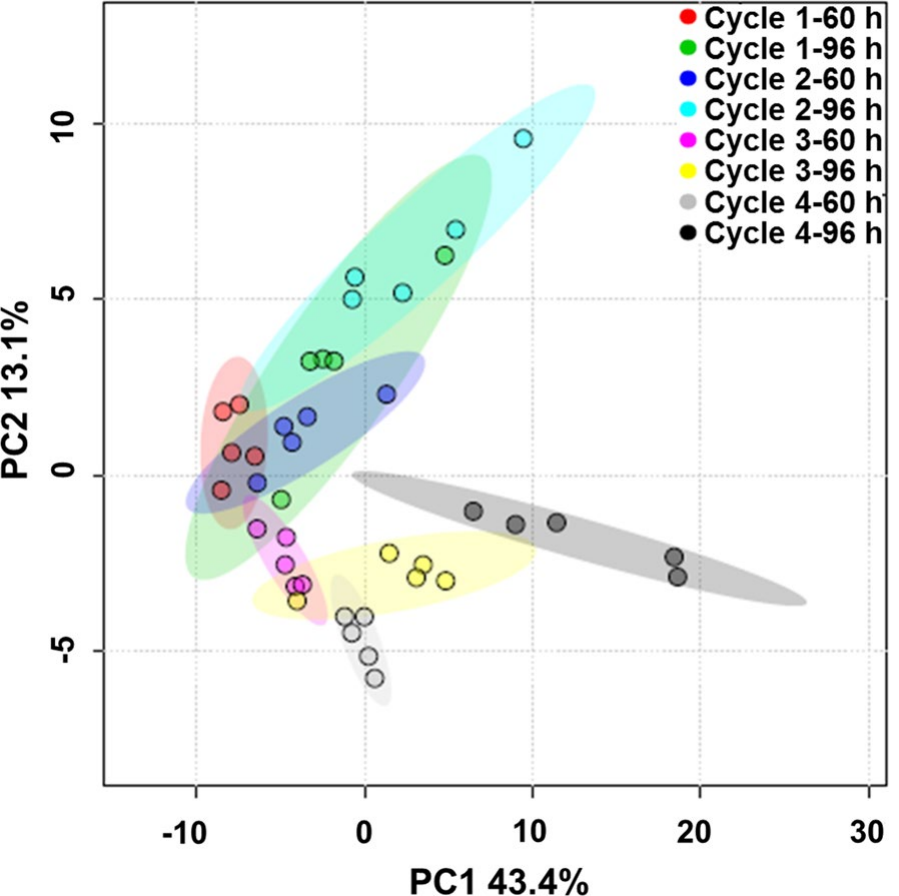
PCA of GC–MS metabolomic profiles of repeated fed-batch cultivation with 4 cycles. The first and second number labeled on top right legends (e.g. cycle 1–60 h) represent the number of cycle and cultivation time, respectively

GC–MS based metabolomic analysis mainly aims at relatively stable metabolites with broader metabolite coverage [20]. In order to further study the mechanisms of attenuated stability of culture system in *C. cohnii* M-1-2 during the repeated fed-batch culture with 4 cycles, GC–MS based metabolomic analysis was carried out. Samples were collected at 60 and 96 h of each cycle during the repeated fed-batch culture. A total of 101 intracellular metabolites were identified in all samples. PCA was used to analyze the overall distribution of the 101 metabolites (Fig. 5). Five biological replicates of each sample gathered well, which indicated that they had good reproducibility. The gradually increased differences at metabolite level for the same time point of different cycles suggested that the changes in cell states were gradually accumulated with the cycle number increased. However, the changes of the relative content of metabolites at the same time point between cycle 1 and 4 were not particularly large, which was consistent with that the DCW change between cycle 1 and 4 of 4.36%.

**Fig. 6:**
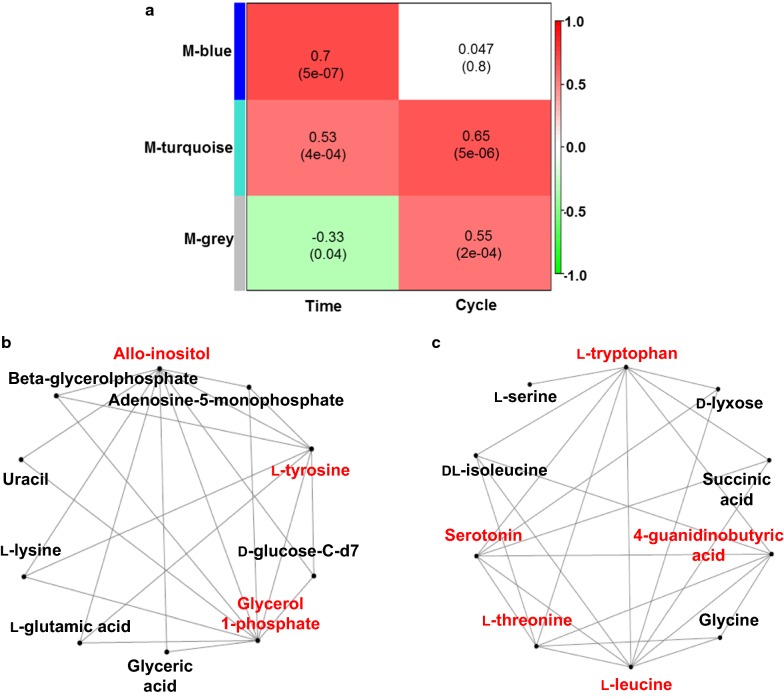
Network visualization of the WGCNA analysis. **a** Module–trait associations. Each row and column representing a module eigen-metabolite or a trait respectively, the first column to time and the second column to cycle; **b** The visualization of top 10 most highly connected metabolites from blue module under the topological overlap threshold at 0.48; **c** The visualization of top 10 most highly connected metabolites from turquoise module under the topological overlap threshold at 0.62

WGCNA is a powerful tool for analyzing omics data [37]. Hub metabolites are highly interconnected metabolites in biological interaction networks, which are considered to have important biological significance [37, 38]. WGCNA was used to identify hub metabolites related to the stability of culture system during the long-term fermentation (Fig. 6). The WGCNA of the GC–MS metabolomic data presented only three modules, suggesting that the changes of metabolites concentration were limited, which was consistent with the PCA results (Fig. 5 and 6a). Using a cutoff of the correlation coefficient *r* > 0.6 (statistical confidence *p* < 0.05), the blue module (*r* = 0.7, correlation *p*-value = 5e^−07^) was found to be positively associated with sampling time points (i.e. 60 and 96 h), while the turquoise module (*r* = 0.65, correlation *p*-value = 5e^−06^) was positively associated with the cycle number. These two modules were further subjected to metabolic network topology analysis and identification of hub metabolites (Fig. 6b, c). Using a cutoff of hub metabolites with connectivity at least 5, allo-inositol, l-tyrosine, and glycerol 1-phosphate located in blue module were found to be positively associated with sampling time points, while l-tryptophan, l-threonine, l-leucine, serotonin, and 4-guanidinobutyric acid located in turquoise module were positively associated with the cycle number, respectively.

Glycerol 1-phosphate is the precursor for triacylglycerol that is the major lipid storage compound in *C. cohnii* [39]. Therefore, association of glycerol 1-phosphate with sampling time points suggested the content of glycerol 1-phosphate was increased at 96 h compared to that at 60 h, which was consistent with the fact that the lipid content at 96 h was higher than at 60 h (Fig. 2). It was also consistent with previous findings that the lipid content (% DCW, *w/w*) of *C. cohnii* was increased with fermentation progressing [9, 40].

The metabolites located at the turquoise module were positively associated with the stability of culture system. The LC–MS based metabolomic analysis showed that PEP was gradually up-regulated at 96 h as the cycle number increased. GC–MS based metabolomic analysis showed l-tryptophan and serotonin were gradually up-regulated as the cycle number increased, suggesting that PEP might be used to synthesize l-tryptophan and serotonin. Tryptophan was also found to be up-regulated under different nitrogen supply conditions for several microbes including *C. cohnii* [3, 41]. For example, the tryptophan was up-regulated under nitrogen excess in *C. cohnii* [3]. However, it is well known that the mechanism involved in tryptophan synthesis is complex and further study is still needed for better understanding the details related to the stability of culture system. In addition to the gradually changed metabolic network, *C. cohnii* M-1-2 might regulate cell metabolism through l-tryptophan, which have been reported to regulate cell metabolism in bacterial cells [42]. For example, tryptophan content in cell was negatively correlated with the growth rate of bacteria [42]. The up-regulation of l-threonine and l-leucine led to further decrease of carbon flow from TCA and glycolytic pathways. Finally, these identified metabolites by LC– and GC–MS could also be used as biomarkers representing the stability of culture system of *C*. *cohnii*. The identification of the relevant biomarkers provided useful information for monitoring the stability of culture system during the repeated fed-batch culture, as well as improving the stability of culture system of *C*. *cohnii* by genetic engineering in the future [43].

## Conclusions

Herein, glucose concentration at 15–27 g/L and 80% medium replacement ratio were found to be suitable for the growth of *C. cohnii* M-1-2 during the repeated fed-batch cultivation. A 4-cycle repeated fed-batch cultivation strategy for DHA production using *C*. *cohnii* was first successfully established and the total DHA productivity was increased by 26.28% compared with the highest DHA productivity of 57.08 mg/L/h reported using *C. cohnii*, taking into consideration of the time for preparing seed culture and fermentor. In addition, gradually decreased nitrogen utilization capacity, and continuous down-regulated glycolysis and TCA cycle were correlated with the decreased stability of culture system. At last, some biomarkers, such as Pyr, Cit, OXA, FUM, l-tryptophan, l-threonine, l-leucine, serotonin, and 4-guanidinobutyric acid, correlated with the stability of the culture system of *C. cohnii* M-1-2 were identified. This study provided not only an efficient and energy-saving strategy for industrial production of DHA, but also important information at metabolite level to monitor the stability of culture system in *C. cohnii* during the long-term repeated fed-batch fermentation. This strategy could also be useful for cultivating other heterotrophic algae to improve productivity and reduce production cost. Moreover, the revealed mechanisms were useful to further optimize production processes for *C. cohnii* and other heterotrophic algae during repeated fed-batch cultivation.

## Methods

### Microorganism and medium

*Crypthecodinium cohnii* M-1-2, a mutant of *C. cohnii* ATCC 30556, was previously isolated by our laboratory [44]. *C. cohnii* M-1-2 is maintained in the basal medium containing 25 g/L sea salt (Sigma-Aldrich, USA), 2 g/L yeast extract (OXOID, UK), and 9 g/L glucose monohydrate (Jiang Tian, China) [20]. The typical fermentation medium includes 25 g/L sea salt, 6 g/L yeast extract, and 27 g/L glucose monohydrate [44].

### Repeated fed-batch culture

The seed culture was prepared as follows: *C*. *cohnii* M-1-2 was inoculated into basal medium, and cultivated for 48 h at 25 °C and 180 rpm; then, 10% (*v/v*) cultures were inoculated into fermentation medium and cultivated for 84 h at 25 °C and 180 rpm. The method to establish individual fermentation cycle was according to our previous publication with minor modification [3]. Briefly, all fed-batch cultures were carried out in a 5-L fermenter (Demei, China); the dissolved oxygen was maintained above 30% air saturation with 1 *vvm* of the air flow level, and automatic regulation of agitation speed; the cultivation temperature was maintained at 25 ± 0.2 °C; and the pH was maintained at 6.5 ± 0.1 with 0.5 M H_2_SO_4_. The antifoam SE-15 was added to eliminate foam when needed. The 50% (*w/v*) yeast extract was used as supplementary nitrogen source between 12 and 96 h of each cycle.

For repeated fed-batch culture, 70–90% of the fermentation broth was removed at 120 h of each cycle and then the same volume of fresh medium was supplemented. 10–30% of the fermentation broth left from the previous fed-batch culture was used as seeds for the next fed-batch culture. To optimize glucose concentration during the fermentation, the initial inoculation volume was 10% (*v/v*) and the replacement ratio of the medium was 90% (*v/v*), respectively, and the glucose concentrations were maintained at 5–15 g/L, 15–27 g/L or 27–45 g/L by feeding with 80% (*w/v*) glucose monohydrate solution. To optimize the replacement ratio, the initial inoculation volume was selected as 10% (*v/v*) with the initial glucose monohydrate concentration 27 g/L, and glucose concentration was maintained at 15–27 g/L. Replacement ratios of 70, 80 and 90% (*v/v*) were evaluated.

### Analysis of DCW, glucose concentration, total lipid content and fatty acids profile

The DCW was measured by gravimetric method after freeze-drying of *C*. *cohnii* cells [3]. The glucose concentration was measured with a Glucose Assay Kit (Biosino, China) [3, 22]. Total lipid content was extracted according to the previous study with a minor modification [45]. Briefly, 25 mg freeze-dried cells of *C*. *cohnii* was extracted by chloroform:methanol (2:1, *v/v*) adding 0.01% butylated hydroxytoluene for three times, and then was washed by 1 M KCl solution and ddH_2_O for one time, respectively. Fatty acids profile were measured according to the previous study [40, 46]. Briefly, freeze-dried cells of *C*. *cohnii* adding with heptadecanoic acid methanol solution (an internal standard), methanol of 3% (*v/v*) sulfuric acid, and chloroform were conducted for reaction at 100 °C for 4 h. The chloroform phase was used to detect after stratification by ddH_2_O.

### LC– and GC–MS based metabolomic analysis

Cell samples collected at 60 h and 96 h for each of the four fermentation cycles of the repeated fed-batch culture were used for LC– and GC–MS metabolomics analyses according to the previous publication [20]. Three and five biological replicates were carried out for LC– and GC–MS metabolomics analysis respectively. The data processing for LC– and GC–MS metabolomics analyses was conducted following the previous study [20]. Metabolomic data was normalized by both cells number and interior control. For LC–MS metabolomic analysis, all standards were purchased from Sigma-Aldrich (MO, USA), and the heatmap was created using MeV 4.9.0 software. For GC–MS metabolomic analysis, principal component analysis (PCA) was created by MetaboAnalyst 4.0 software available at https://www.metaboanalyst.ca/.

### Weighted gene co-expression network analysis (WGCNA)

WGCNA was carried out for GC–MS metabolomic analysis. The method for network construction, module identification, and the relationship between modules and external conditions/traits followed the standard metabolomic analysis procedure of WGCNA described previously [37]. All metabolomic analyses were firstly converted into z-score for WGCNA construction. To identify the biologically significant modules with cycle and time through WGCNA, the value of the correlation coefficient *r* was set more than 0.6 and the statistical confidence *p*-value was set less than 0.05 [20]. Further downstream analysis for associated modules of biological significance was carried out to identify potential hub metabolites. Visualization of 10 most highly connected metabolites was conducted using the Cytoscape software [47]. Metabolites with a number of connections greater than or equal to 5 with other metabolites were considered as hub metabolites [20].

### Statistical analysis

All measurements of DCW, glucose concentration, total lipid content and fatty acids profile were performed in at least three analytical replicates. All data were expressed in the form of mean values ± standard deviations. Student’s *T*-test was used to evaluate the data (*p < 0.05, **p < 0.01, ***p < 0.001).

## Data Availability

The datasets used and analyzed during the current study are available from the corresponding author on reasonable request.

## Abbreviations

DHA: Docosahexaenoic acid
DCW: Dry cell weight
PUFAs: Polyunsaturated fatty acids
TFAs: Total fatty acids
EPA: Eicosapentaenoic acid
ARA: Arachidonic acid
PCA: Principal component analysis
TCA: Tricarboxylic acid
ATP: Adenosine triphosphate
F6P: Fructose 6-phosphate
Suc: Succinate
ADP-Glu: Adenosine diphosphoglucose
R5P: Ribose 5-phosphate
1,3-PG: 1,3-Bisphosphoglyceric acid
NAD: Nicotinamide adenine dinucleotide
Mal: Malate
ADP: Adenosine diphosphate
Asp: Aspartic acid
GAP: Glyceraldehyde 3-phosphate
Glu: Glutamic acid
AMP: Adenosine monophosphate
Phe: Phenylalanine
NADPH: Nicotinamide adenine dinucleotide phosphate
RiBP: Ribulose 1,5-bisphosphate
3-PG: 3-Phosphoglyceric acid
PEP: Phosphoenolpyruvic acid
NADP: Oxidized form of nicotinamide adenine dinucleotide phosphate
AKG: 2-Oxoglutaric acid
CoA: Coenzyme A
FUM: Fumaric acid
OXA: Oxaloacetic acid
Cit: Citric acid
Pyr: Pyruvate
AcCoA: Acetyl coenzyme A
FBP: Fructose 1,6-bisphosphate
